# Automated vs. human evaluation of corneal staining

**DOI:** 10.1007/s00417-022-05574-0

**Published:** 2022-03-31

**Authors:** R. Kourukmas, M. Roth, G. Geerling

**Affiliations:** Department of Ophthalmology, Heinrich-Heine University Düsseldorf, Moorenstr. 5 40225, Düsseldorf, Germany

**Keywords:** Cornea, Dry eye disease, Grading, Image analysis

## Abstract

**Background and purpose:**

Corneal fluorescein staining is one of the most important diagnostic tests in dry eye disease (DED). Nevertheless, the result of this examination is depending on the grader. So far, there is no method for an automated quantification of corneal staining commercially available. Aim of this study was to develop a software-assisted grading algorithm and to compare it with a group of human graders with variable clinical experience in patients with DED.

**Methods:**

Fifty images of eyes stained with 2 µl of 2% fluorescein presenting different severity of superficial punctate keratopathy in patients with DED were taken under standardized conditions. An algorithm for detecting and counting superficial punctate keratitis was developed using ImageJ with a training dataset of 20 randomly picked images. Then, the test dataset of 30 images was analyzed (1) by the ImageJ algorithm and (2) by 22 graders, all ophthalmologists with different levels of experience. All graders evaluated the images using the Oxford grading scheme for corneal staining at baseline and after 6–8 weeks. Intrarater agreement was also evaluated by adding a mirrored version of all original images into the set of images during the 2nd grading.

**Results:**

The count of particles detected by the algorithm correlated significantly (*n* = 30; *p* < 0.01) with the estimated true Oxford grade (Sr = 0,91). Overall human graders showed only moderate intrarater agreement (*K* = 0,426), while software-assisted grading was always the same (*K* = 1,0). Little difference was found between specialists and non-specialists in terms of intrarater agreement (*K* = 0,436 specialists; *K* = 0,417 non-specialists). The highest interrater agreement was seen with 75,6% in the most experienced grader, a cornea specialist with 29 years of experience, and the lowest was seen in a resident with 25,6% who had only 2 years of experience.

**Conclusion:**

The variance in human grading of corneal staining - if only small - is likely to have only little impact on clinical management and thus seems to be acceptable. While human graders give results sufficient for clinical application, software-assisted grading of corneal staining ensures higher consistency and thus is preferrable for re-evaluating patients, e.g., in clinical trials.
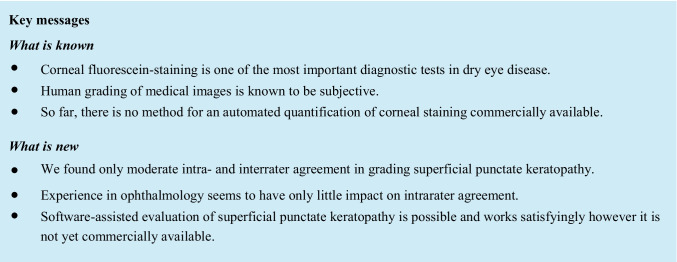

**Supplementary Information:**

The online version contains supplementary material available at 10.1007/s00417-022-05574-0.

## Introduction

Fluorescein staining is one of the most important diagnostic tests for clinical and research purposes in dry eye disease (DED) [1]. While more and more examinations are being assisted by computers (optical coherence tomography, corneal topography, wavefront analyses) in the last decades, objective methods for an automated quantification of corneal staining have been developed, but are not yet commercially available [2–5]. The aim of our study was to examine if software-assisted grading is superior to human grading in accuracy and consistency. The problem of high intra- and interrater error in human grading of medical images is a known problem in ophthalmology and other fields of medicine [6–9]. In particular human grading of corneal staining with different scores is known to be subjective and lacks reproducibility [10]. There are 41 different grading scales to evaluate the ocular surface in humans, of which 18 are for grading corneal and/or conjunctival staining [11]. The choice of the grading scale has effect on both sensitivity and consistency. While fewer steps within a grading system lead to good repeatability, they mostly lack sensitivity [12–14]. For this reason, a grading system with 0–100 steps was developed [15]. Higher numbers of possible grades on the other hand tend to produce inconsistent results and might be biased by the well-known problem that human graders tend to choose numbers that can be divided by five more often than others what again reduces the amount of steps and therefore the sensitivity [15, 16]. The Oxford scale consisting of grades from 0 to 5 is one of the most commonly used grading scales for corneal staining. Considering the diverse nature of superficial punctate keratitis, a 0–5 gradation seems relatively coarse. Therefore, an automated grading system which is not limited to a specific scale would be favorable.

## Material and methods

### Acquisition of corneal images

Images of 50 eyes with different grades of dry eye disease were taken under standardized conditions. Two microliter of 2% fluorescein were instilled into the lower fornix with a 2 µl Eppendorf Pipette. After 30 s, the images were acquired with a Canon camera model DS126251 attached to a Haag-Streit photo slit lamp model 900.8.2.0165 with diffuse lightning, yellow filter and 10 × enlargement in a completely dark room, and saved in red–green–blue-format (RGB). Twenty of these images were used as a training set to develop the algorithm and 30 as training dataset for comparison with the human graders.

### ImageJ algorithm for automated quantification of corneal staining

ImageJ, the most common software for image analysis and processing in biological research, was used for automated quantification of corneal staining [17]. All functions used for preprocessing and analyzing the images are commonly used in scientific image analysis. “Auto-threshold” was used to detect particles by separating the images into a foreground and background depending on differences of intensity. The background was eliminated, leaving the foreground with the “objects of interest” for quantification. Many different auto-threshold methods are available. Comparing the different methods using the training dataset, we found best conformity with “triangle-white” to isolate and count particles without having a large number of false positives from artifacts [18]. Next, to exclude possible artifacts like tear film or mucus, size and circularity of the “objects of interest”, i.e., positive epithelial staining, had to be specified. Following repetitive assessment using the training-dataset, particles bigger than 200 pixels or with circularity below 0,7 were eliminated. After defining those prerequisites, a macro was developed, that executed the following steps, when the cornea was marked manually as region of interest (ROI).

**Fig. 1:**
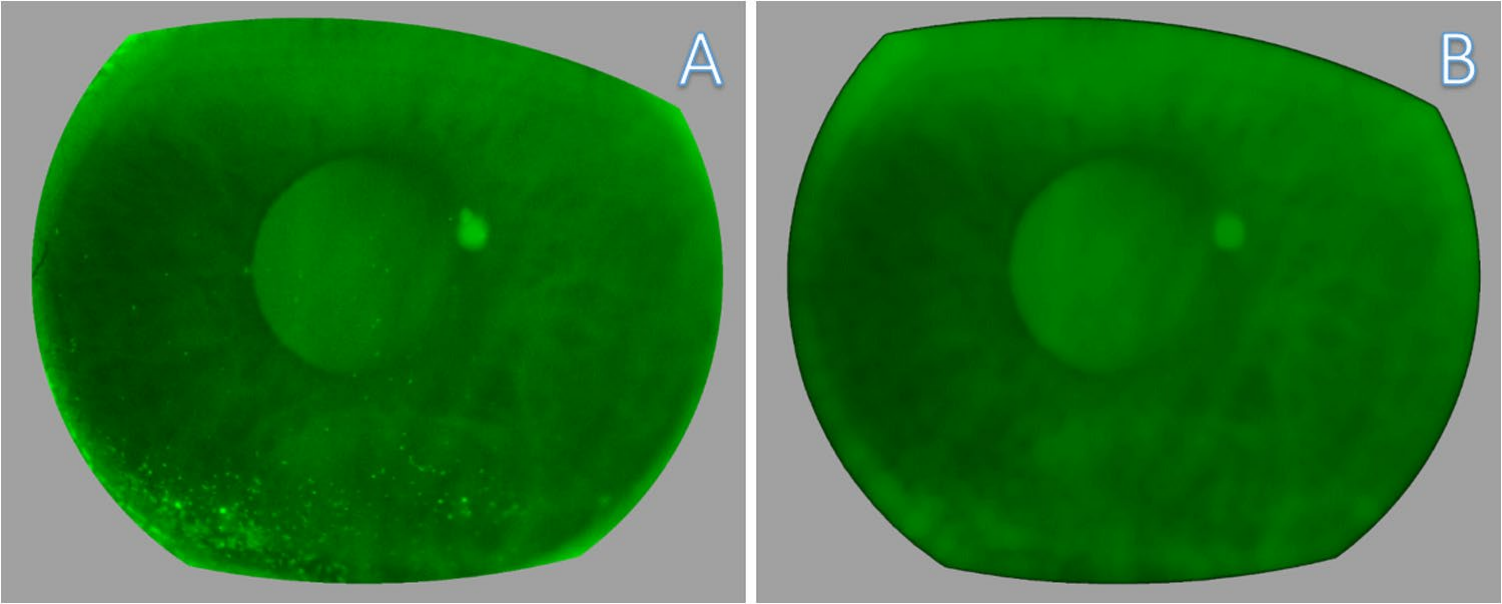
**A** Original image and **B** artificial pseudo-background

Preprocessing: The green channel of the RGB image was isolated and transformed into 8 bit format. With ImageJ embedded automatic contrast enhancement, the distribution of intensities became wider for better separation. Convoluted background subtraction with a radius of 14 pixels and a Gaussian blur with a sigma of 2 pixels were used to generate a so-called pseudo-background (Fig. 1) which was then subtracted from the main image to remove artifacts and background structures such as the iris, pupil, or tear film artifacts.

**Fig. 2:**
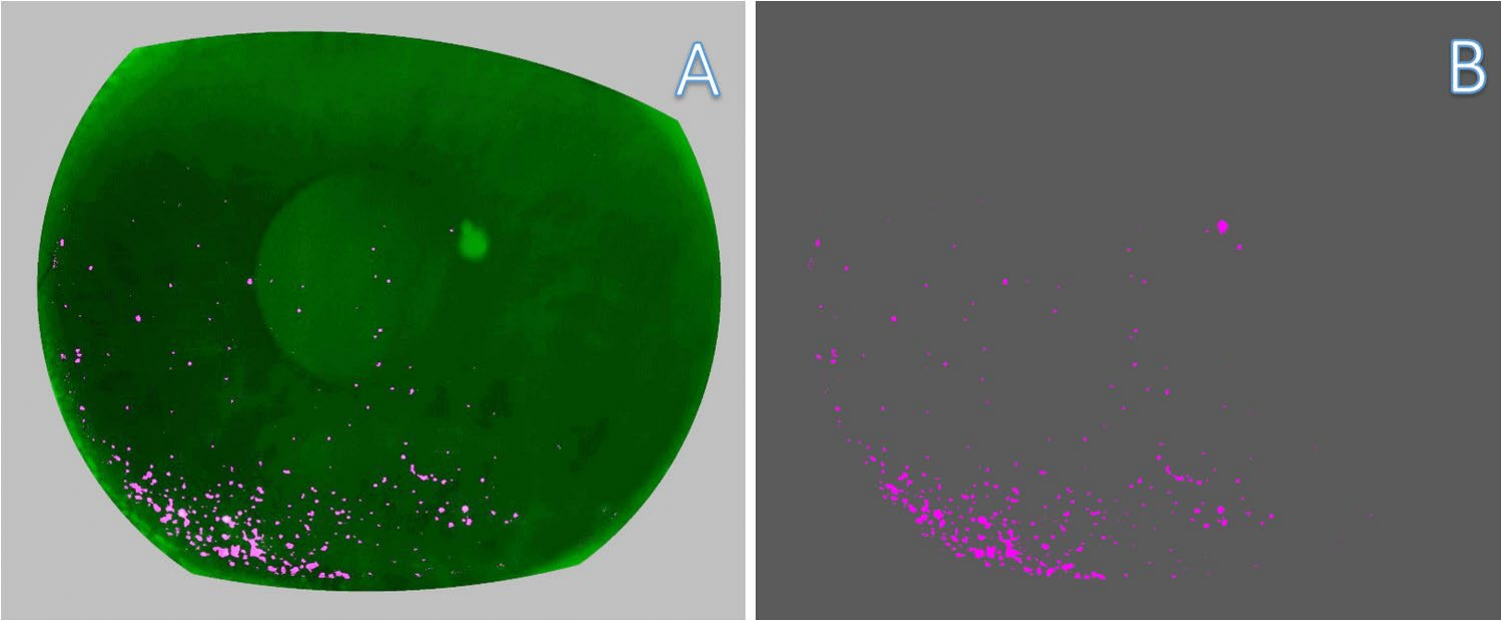
**A** Detected corneal staining overlay and **B** particle mask. Brightness was adjusted for illustration

Analysis: Auto-threshold triangle-white technique was used to isolate particles from remaining noise. Then, a binary mask was created, showing only two intensities (1 = positive staining; 0 = no staining) (Fig. 2). Finally, the number of particles with the defined size and circularity was counted. Execution of this macro takes approximately 3–5 s per image on an average desktop computer. For the exact script of the macro, see Supplements.

### Human grading of corneal staining

A cohort of 22 graders, 9 board certified ophthalmologists, 11 residents, and 2 medical students with less than 1 year of experience of the Department of Ophthalmology, University Hosptial Düsseldorf, were asked to grade the full test set. Grading was performed according to the Oxford classification for corneal staining under standardized conditions with a tablet computer (Samsung® Galaxy Tab S2) with a high-quality display and full brightness in a completely dark room [19].

The Oxford scheme with sample graphics was displayed to the graders throughout the entire grading process on the same screen, below the image that was to be graded. After 6–8 weeks, all participants graded the identical 30 images twice again, once as original and once mirrored horizontally, without previously being informed about this second grading and the fact that the identical images were used and had been mirrored. Software results were then compared to human grading. There was no time limit for the graders to complete the grading, but the full test set had to be graded in a single episode.

### Statistics

SPSS version 27 (IBM, USA, NY, Armonk) was used for statistical analysis. Cohens-Kappa and Fleiss-Kappa were used to evaluate intra- and interrater reliability. For interpretation of *K*-values, Landis and Koch Table were used. The software-assisted evaluation (measured in number of particles) was compared to the most frequent picked Oxford grade (estimated true) using Spearman’s rank correlation. A *p*-value below 0,05 was regarded as statistically significant.

## Results

### Interrater agreement

In the first grading episode, human interrater agreement was *K* = 0,462 and thus moderate between all graders. The result was the same, when all human ratings were analyzed together, i.e., all gradings from the first and second round of gradings (*K* = 0,426). Table 1 shows the deviation for every grader from the estimated true Oxford grade. The highest agreement with the estimated true Oxford grade was seen in the most experienced grader in 75,56% of all cases. Deviation by more than one Oxford grade from the estimated true Oxford grade was seen in 18 of 22 graders. While there was a maximal deviation of 4 Oxford grades in one case of a non-specialist, deviation of 3 Oxford grades was seen in three participants (7 cases). Resident number 8 showed a deviation of three Oxford grades in three cases and selected the estimated true Oxford grade only in 25,6% of all cases, as one of the students.

**Table 1:** Distribution of deviation from the estimated true Oxford grade for every grader in percentage

Grader	Deviation of grading from estimated true Oxford scale				
	0	1	2	3	4
Specialist 1	75,56%	22,22%	1,11%	-	-
Specialist 2	73,33%	26,67%	-	-	-
Specialist 3	42,22%	48,89%	4,44%	-	-
Specialist 4	54,44%	32,22%	4,44%	-	1,11%
Specialist 5	70,00%	30,00%	-	-	-
Specialist 6	71,11%	28,89%	-	-	-
Specialist 7	50,00%	37,78%	4,44%	1,11%	-
Specialist 8	67,78%	27,78%	2,22%	-	-
Specialist 9	72,22%	23,33%	2,22%	-	-
Resident 1	64,44%	26,67%	4,44%	-	-
Resident 2	62,22%	33,33%	2,22%	-	-
Resident 3	58,89%	34,44%	3,33%	-	-
Resident 4	71,11%	26,67%	1,11%	-	-
Resident 5	61,11%	34,44%	2,22%	-	-
Resident 6	67,78%	30,00%	1,11%	-	-
Resident 7	66,67%	28,89%	2,22%	-	-
Resident 8	73,33%	26,67%	-	-	-
Resident 9	25,56%	46,67%	8,89%	3,33%	-
Resident 10	57,78%	35,56%	3,33%	-	-
Resident 11	45,56%	40,00%	5,56%	1,11%	-
Student 1	25,56%	54,44%	6,67%	2,22%	-
Student 2	73,33%	24,44%	1,11%	-	-

### Intrarater agreement

**Fig. 3:**
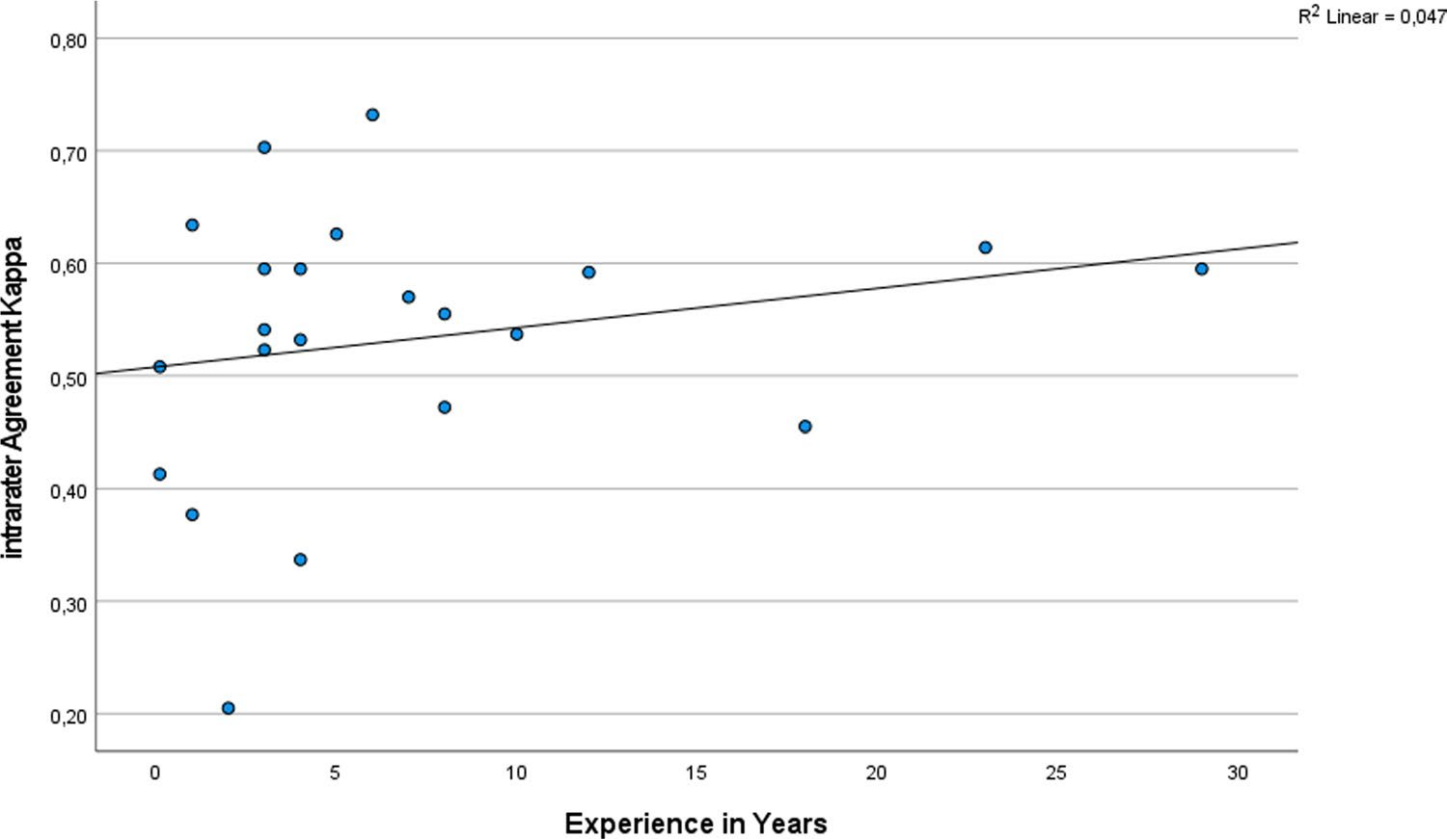
Total intrarater agreement with experience in years

All *K*-values for every grader are listed in Table 2. Specialists and non-specialists showed “moderate” intrarater agreement, but specialists were slightly more consistent than non-specialists (*K* = 0,436 specialists; *K* = 0,417 non-specialists). The most experienced grader, a cornea specialist with circa 30 years of experience was most consistent in his grading with an “almost perfect” agreement of *K* = 0,831 for grading the native and the mirrored images in the second grading episode. The lowest intrarater agreement was found in a resident with *K* = 0,155 (“slight agreement”) for re-evaluation after 6–8 weeks. Figure 3 shows the total intrarater agreement in relation to years of experience in ophthalmology.

**Table 2:**
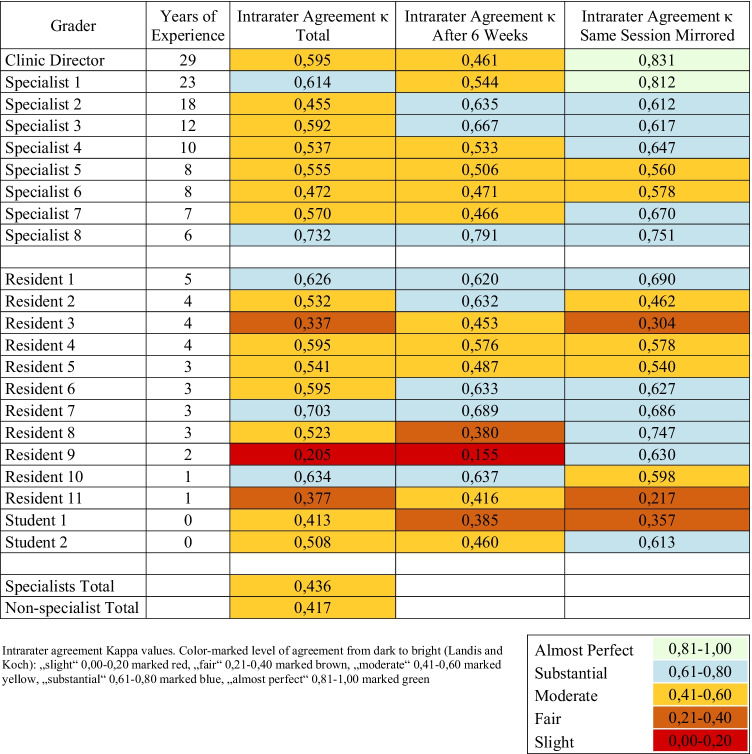
Intrarater agreement: kappa values for all graders

### Automated grading

**Fig. 4:**
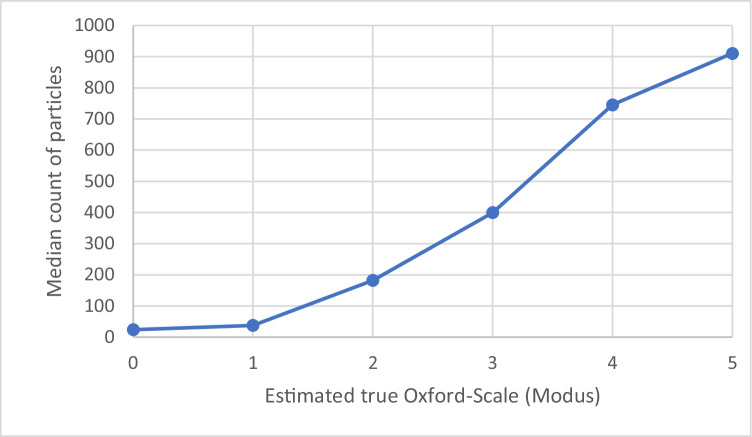
Median count of particles detected by software in dependence to the estimated true Oxford scale (modus) for all images

Software-assisted grading was identical after 6 weeks and not affected by mirroring the images. The count of particles detected by the algorithm correlated significantly (*n* = 30; *p* < 0.01) with the estimated true Oxford grade (Sr = 0,91) (see Fig. 4).

## Discussion

Experience is a well-known denominator of grading precision in ophthalmology [20]. Although only little difference was found between specialists and non-specialists overall, Fig. 1 shows that lower intrarater agreement is seen in the less experienced graders. Nevertheless, our results show that even highly experienced graders are not as consistent as a software-assisted grading method. Highest inconsistency (low intrarater agreement) was found when pictures were regraded after 6–8 weeks (*K* = 0,461). This temporal intrarater agreement is also known as temporal drift or grade-regrade-agreement [21]. Ebenezer et al. have worked on grading of retinopathy of prematurity and used a particular temporal drift sample of 25 images that were regraded at three different points of time and found strong variety in intrarater agreement over time ranging from 0.57 to 0.94 [22]. This variation over time in human grading especially might become a problem in study settings, where reliable data needs to be gathered.

Nichols et al. investigated repeatability of several DED parameters at two time points including only one grader and found poor to moderate intrarater agreement for corneal fluorescein staining [23].

Rasmussen et al. investigated human grading of corneal and conjunctival staining on the slit lamp in 11 physicians with van Bijstervald score (vBS) and the ocular staining score (OSS) and found moderate to good intrarater agreement with intraclass correlation coefficient (ICC) of 0.77 for the vBS and 0.74 for the OSS [10]. It should be mentioned that the study mainly focused on the comparison between vBS and OSS; thus, only a small number of individuals (20 out of total 994) were invited for a second examination, and only nine were re-evaluated by the same physician.

Beyond the limited intrarater agreement, in a real-world setting of a busy clinic with rising number of follow-up visits, a patient is likely to be examined by different individuals adding interrater error. Unlike intrarater agreement, interrater agreement is difficult to investigate because there is no certainty about the true Oxford grade of an image. Furthermore, Fleiss-Kappa measures whether the grading is identical between the two time points but do not quantify a possible deviation. While grading corneal staining referring to the Oxford scheme is a method of comparing a slit lamp image with a graphic scheme, it can be assumed that the true Oxford grade for a picture is the one that was most frequently picked. Therefore, modus was chosen for the estimated true Oxford grade in our study. Rasmussen et al. still found significant variation between human graders, although all participants had undergone a particular training before. As every participant graded a different subject, the results are difficult to compare to our cohort study.

Rodriguez et al. found a mean concordance correlation coefficient (CCC) of 0.882 between three human graders grading 54 images in the Ora Calibra Fluorescein Staining Scale, what can be considered good reliability [24]. CCC was not calculated for our cohort, because it is more suitable to assess agreement between two, but not multiple graders. Amparo et al. tested interrater agreement in four clinicians grading 61 images using the National Eye Institute/Industry (NEI) grading scale and gathered ICC of only 0.65, what is considered moderate interrater agreement [25]. For better comparison, we calculated ICC from our cohort and gathered 0.994 what is considered excellent reliability. We want to put this value into perspective as we found only moderate agreement with Fleiss-Kappa and high deviation of 3 or 4 Oxford grades could be found between graders for both, specialists, and non-specialists. This difference can be explained by the fact that Fleiss-Kappa, as mentioned earlier, only measures whether the same grade has been chosen and ICC also respects the level of disagreement between two Oxford grades. Therefore, we think neither ICC nor Fleiss-Kappa solemnly can represent the true agreement between graders, as it is necessary for clinical practice or study settings.

While time between two gradings can especially influence the intrarater-results, there are some parameters that might influence both intra- and interrater grading. First, conditions in real live grading could vary depending on possible fluctuation in light situations or use of different slit lamp settings. Second, also the patient reported symptoms or conjunctival hyperaemia might bias a human grader in his evaluation. In addition, there may be differences in grading photographed slit lamp images versus live grading [26].

In contrast to human grading, a computer-based evaluation is not bound to a specific scale but simply counts predefined affected areas. Our algorithm showed proper correlation with the Oxford grades (Sr = 0,91; *n* = 30; *p* < 0.01). The previously mentioned groups have developed similar algorithms for corneal staining and compared the results to human grading. Rodriguez et al. used an algorithm programmed with OpenCV© (Open Source Computer Vision Library) and focused on the inferior corneal staining as region of interest and used the Ora Calibra Staining Scale®, a logarithmic scale for the number of counted particles [24]. The software-based grading was compared to human grading results. In their study, the agreement between human and software-assisted grading was high (*R* = 0.89) [24].

Amparo et al. analyzed the complete corneal area similar to us but used the National Eye Institute/Industry (NEI) grading scale and compared the results of human grading with those of an algorithm programmed in ImageJ [25]. They reported a significant correlation between their software-assisted method and human grading (*R* = 0.72) [25].

Chun et al. compared the grading of two independent clinicians using the Oxford scheme and the National Eye Institute/Industry (NEI)-recommended guidelines to a software-assisted method programmed in Microsoft Visual C +  + and Open CV©. They achieved high correlation between the software-based grading and both human grading scores (Oxford scheme: *R* = 0.85; NEI: *R* = 0.903) [27].

While the above-mentioned groups have achieved similar results to our cohort, the main difference and novelty in our study are the large number of human graders with different levels of experience using the grade-regrade method that allows the best possible comparison between them. Overall, as shown in our work and the other studies mentioned above, software-based grading achieves sufficient results with precision at least as accurate in comparison to human grading. Comparison between the different groups in case of precision of the algorithm is difficult because there is difference in the selected region of interest and the chosen grading score. Although software-assisted grading might be challenged, e.g., by confluent staining, by refining the techniques and algorithms, and, e.g., by application of deep learning, we think it will still be superior to a pictogram-based grading system in the future. Besides precision, another advantage of a computer-based evaluation is its consistency. Similar to the results of the grading of corneal staining, software-assisted grading of ocular redness or conjunctival lissamaine green staining has been shown to be superior compared to human evaluation [16, 28–30].

Also clinical studies would probably benefit from such a technique, because corneal fluorescein staining often is considered an important endpoint, e.g., in the SANSIK study, a multicenter phase III study for cyclosporine-A eye drops [31]. Especially in a multicenter setup with numerous graders, a more objective method would be favorable. Furthermore such a method could be used for a more exact (sub-)staging of several corneal conditions, e.g., like neurotrophic keratopathy [32]. While the benefit of a software-assisted grading system in clinical studies is obvious, it should be noted that in a clinical setting small deviation in the grading of corneal staining is acceptable and does probably rarely lead to changes in treatment. A deviation of 3–4 Oxford grades as we found in some cases though cannot be considered as negligible.

## Conclusion

High inter- and intrarater bias has been seen in human grading of corneal fluoresceine staining. While accuracy of human grading may be considered sufficient, it lacks intra- and interrater consistency. Although the measured inconsistency is likely to have little impact on clinical management and outcome, an objective method would be beneficial for study settings and development of more precise staging schemes of anterior eye diseases.

## Supplementary Information

Below is the link to the electronic supplementary material.

Supplementary file1 (DOCX 28 KB)

## Data Availability

Not applicable.

## Code availability

Algorithm available in Supplements.
