# Supplementary material for: Automated vs. human evaluation of corneal staining
Source: Graefes Arch Clin Exp Ophthalmol. 2022 Mar 31;260(8):2605–12. doi: 10.1007/s00417-022-05574-0 (PMC9325848; doi:10.1007/s00417-022-05574-0)
Supplement: Supplementary file 1 — Supplementary file1 (DOCX 28 KB) [file 417_2022_5574_MOESM1_ESM.docx]

**Supplement**

Macro for preprocessing and analysis

*run("Delete Slice", "delete=channel");*

*run("Stack to Images");*

*rename("blue");*

*selectWindow("blue");*

*close();*

*rename("green");*

*run("8-bit");*

*run("Enhance Contrast...", "saturated=0.01");*

*run("Convoluted Background Subtraction", "convolution=Gaussian radius=14");*

*run("Gaussian Blur...", "sigma=2");*

*run("Auto Threshold", "method=Triangle white");*

*setOption("BlackBackground", true);*

*run("Convert to Mask");*

*run("Analyze Particles...", "size=0-200 pixel circularity=0.7-1.00 show=[Masks] display exclude summarize in_situ");*
